# How New Developments in Pharmacology Receptor Theory Are Changing (Our Understanding of) Hypertension Therapy

**DOI:** 10.1093/ajh/hpad121

**Published:** 2023-12-27

**Authors:** Stephanie W Watts, Raymond R Townsend, Richard R Neubig

**Affiliations:** Department of Pharmacology and Toxicology, College of Osteopathic Medicine, Michigan State University, East Lansing, Michigan 48824-131, USA; Department of Nephrology and Hypertension, University of Pennsylvania Medical Center, Philadelphia, Pennsylvania 19104, USA; Department of Pharmacology and Toxicology, College of Osteopathic Medicine, Michigan State University, East Lansing, Michigan 48824-131, USA

**Keywords:** agonist, antagonist, blood pressure, constitutive activity, drug–receptor interaction, drug receptor theory, hypertension, inverse agonist

## Abstract

**Background:**

Many hypertension therapeutics were developed prior to major advances in drug receptor theory. Moreover, newer drugs may take advantage of some of the newly understood modalities of receptor function.

**Goal:**

The goal of this review is to provide an up-to-date summary of drug receptor theory. This is followed by a discussion of the drug classes recognized for treating hypertension to which new concepts in receptor theory apply.

**Results:**

We raise ideas for mechanisms of potential new antihypertensive drugs and whether they may take advantage of new theories in drug–receptor interaction.

The readers of this journal have been and are the force to treat the disease of hypertension. It has been for only ~120 years that high blood pressure has been recognized as a clinically meaningful finding, as determined by insurance and actuarial companies.^1^ Further, at least some in the medical community were skeptical of the *necessity* to treat hypertension. The following quote is attributed to Dr. Paul Dudley White, an important Boston Cardiologist: “Hypertension may be an important compensatory mechanism which should not be tampered with, even were it certain that we could control it.”^2^ Significant research that started in the 1940s identified that control of blood pressure was key to extending lifespan. Myriad treatments have been used, including surgical interventions. Pharmacology, however, provides a versatile, less invasive means by which to treat the life-long problem of hypertension.

The use of pharmaceutics in the treatment of hypertension began with the success of the compound tetraethylammonium chloride (TEA) to reduce blood pressure.^3^ This quaternary ammonium salt was recognized to inhibit the transmission of nerve impulses. Injected intravenously, it reduced arterial pressure. The first successful (oral) agent to treat hypertension was chlorothiazide as a diuretic, in 1957.^4^ This drug is still used today. Figure 1 presents a “work in progress” timeline for antihypertensive medications, many of which are described below. It shares either specific drugs or drug classes. The empty boxes are present for reasons also discussed below. As shown in Figure 1, the discovery of antihypertensive medications was extensive in the 1950s–1970s but has been sporadic ever since.

**Figure 1.**
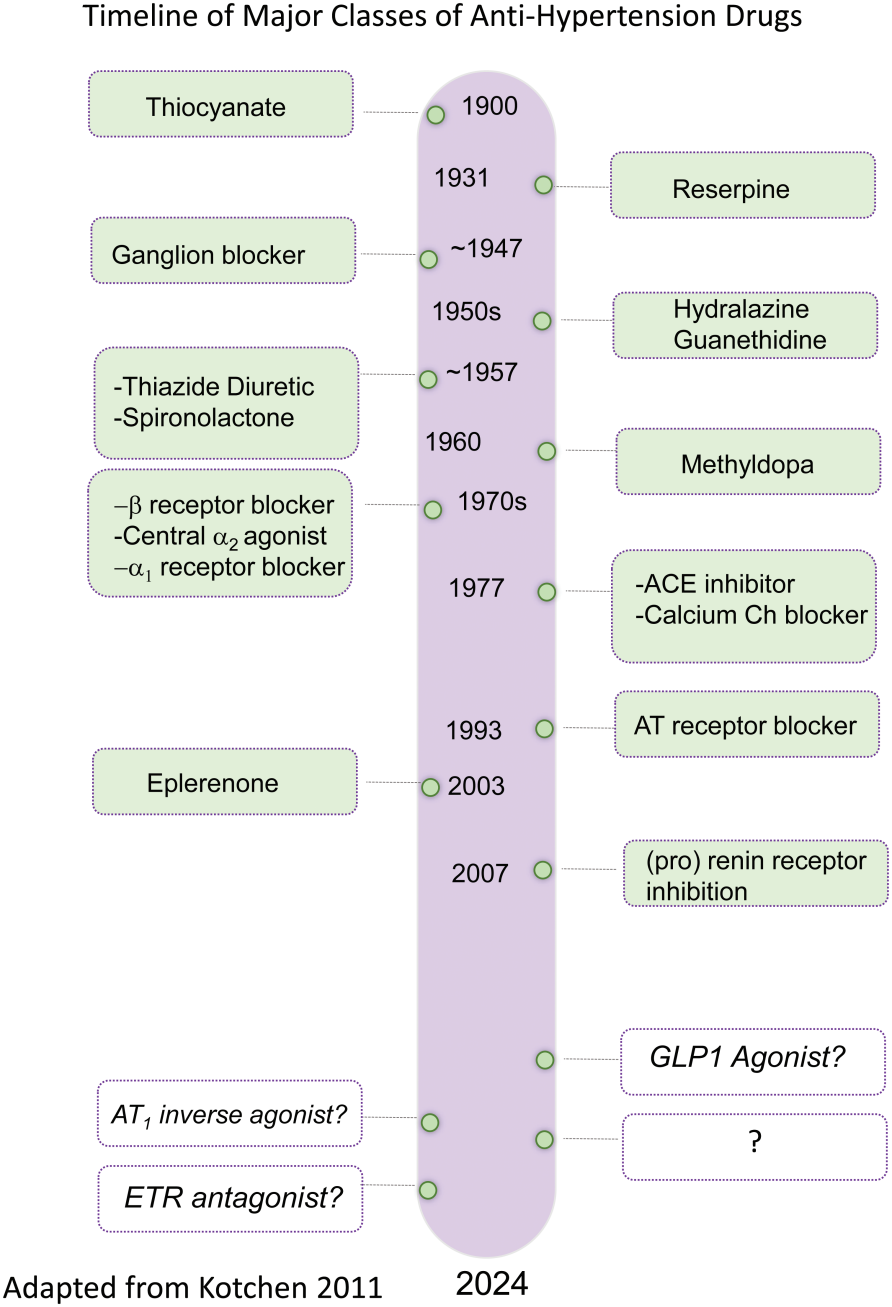
Time line of development/first use of antihypertensive therapies. Both individual and drug classes are named in the light green shaded boxes. The colorless boxes have the potential to be used as antihypertensives or to be discovered. Adapted from Kotchen 2011.

Since the purposeful use of chlorothiazide in 1957, knowledge of the functions of **receptors**—the functional targets of drugs—has advanced mightily. Figure 2 depicts the four (4) major classes of receptors recognized by pharmacologists: G protein-coupled receptors (GPCRs), ion channel receptors, enzyme-linked receptors, and nuclear receptors. These four classes of receptors were not formally recognized at the time of the initial use of chlorothiazide. New knowledge that receptors are biological targets for drugs provided the ability to take advantage of the many ways that receptors function, as well as allowing the field to capitalize on differences and similarities. Advances in drug receptor theory, decade upon decade, have enabled further advances in antihypertensive drug discovery.

**Figure 2.**
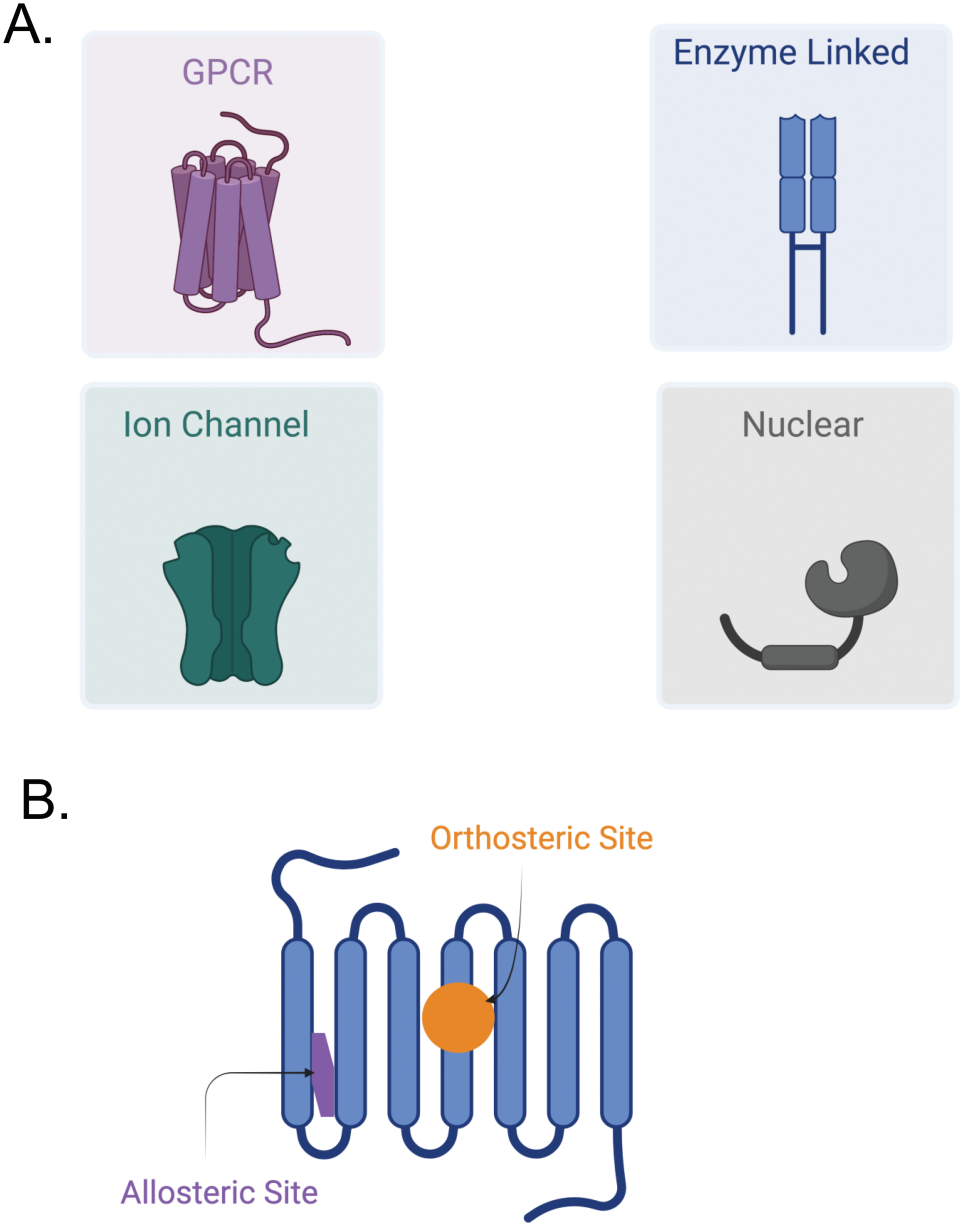
(**a**) Depiction of the four (4) major classes of receptors recognized by pharmacologists. (**b**) Depiction of the difference in site of action of an orthosteric agonist (orange circle) and allosteric modulator (purple shape).

The goal of this review is to provide an up-to-date summary of drug receptor theory and focus on these new theories as they apply to receptor-targeted antihypertensive drugs. A discussion of general receptor theory is followed by a discussion of relevant drug classes recognized for treating hypertension. Both known mechanisms of actions and potential new functions of drug classes and/or particular drugs will be discussed, based on new drug receptor theories. We end with a discussion of potential new antihypertensive drugs and how they take advantage of new theories in drug–receptor interaction.

## Drug receptor theory

Despite very different signal transduction mechanisms, all four classes of recognized receptors possess common pharmacological characteristics that are exploited for therapeutic advantage. The most fundamental property of a drug (or ligand) interacting with a receptor is its affinity or how tightly it binds to the receptor. The second property is what it does to change the function of the receptor, and its efficacy; this property results in a change in biological activity mediated by receptor activation. While the existence of drugs which activate receptors, agonists, and those that inhibit receptor function, antagonists, has been known for over 100 years, recent advances in understanding subtle differences between different drugs acting on a single receptor open a new horizon for improved pharmacotherapeutics of hypertension.

Here we give a high-level overview of modern drug receptor theory as it stands today and where current and future hypertension therapies fit into our new understanding of receptor function. For those more interested in modern drug receptor theory, we recommend “A Pharmacology Primer.”^5^ Quantitative drug receptor theory, published by AJ Clark,^6^ began to develop about 10 years prior to use of TEA. As increased understanding of drugs used to modify blood pressure occurred, so too was drug receptor theory refined. The work of Ahlquist in 1948 is specifically important to the field of hypertension in defining that there were different types of adrenergic receptors.^7^ In figures, we use a GPCR or a generic receptor for illustration. A majority of the work done to construct the above theories involved GPCRs.^8^ This is reasonable given that GPCRs represent the primary target of 34% of FDA-approved drugs.^9^

## Affinity: common ground for drugs

The basis of all drug action is drug binding to (i.e., having affinity for) its target. This concept was propounded by Ehrlich in 1913 based on Paracelsus “*corpora nonagunt nisi fixata*” [agents only work when they are bound^10]^. Affinity means that the drug, be it a small molecule, a protein, or a large antibody interacts physically with some part of the receptor, typically a protein. In the case of an agonist drug, those interactions change the function of the receptor. Receptors can be at the membrane of the cell or internal to the cell in the cytoplasm, on organelles, or in the nucleus. The drug interacts with a specific binding site on its target receptor to cause an effect (Figure 2b). Drug binding can have various levels of strength or affinity, determined by the particular types of interactions or forces: van der Waals interactions, hydrogen bonding, dipole to dipole, ionic binding, or covalent/irreversible binding. This determines the affinity that a drug has for a receptor and contributes to the drug’s potency (see below).

Receptors may have multiple sites at which drugs can bind. The primary binding site is that at which the physiological agonist binds to activate the receptor. It is called the **orthosteric** binding site (Figure 2b). Many receptors have other binding sites to which a drug can bind to influence receptor function. These are called **allosteric** binding sites and while they do not overlap physically with the orthosteric site on the receptor, drug binding at the two sites can influence each other in the overall actions of the receptor (Figure 2b).

## Agonism: POTENCY

**Figure 3.**
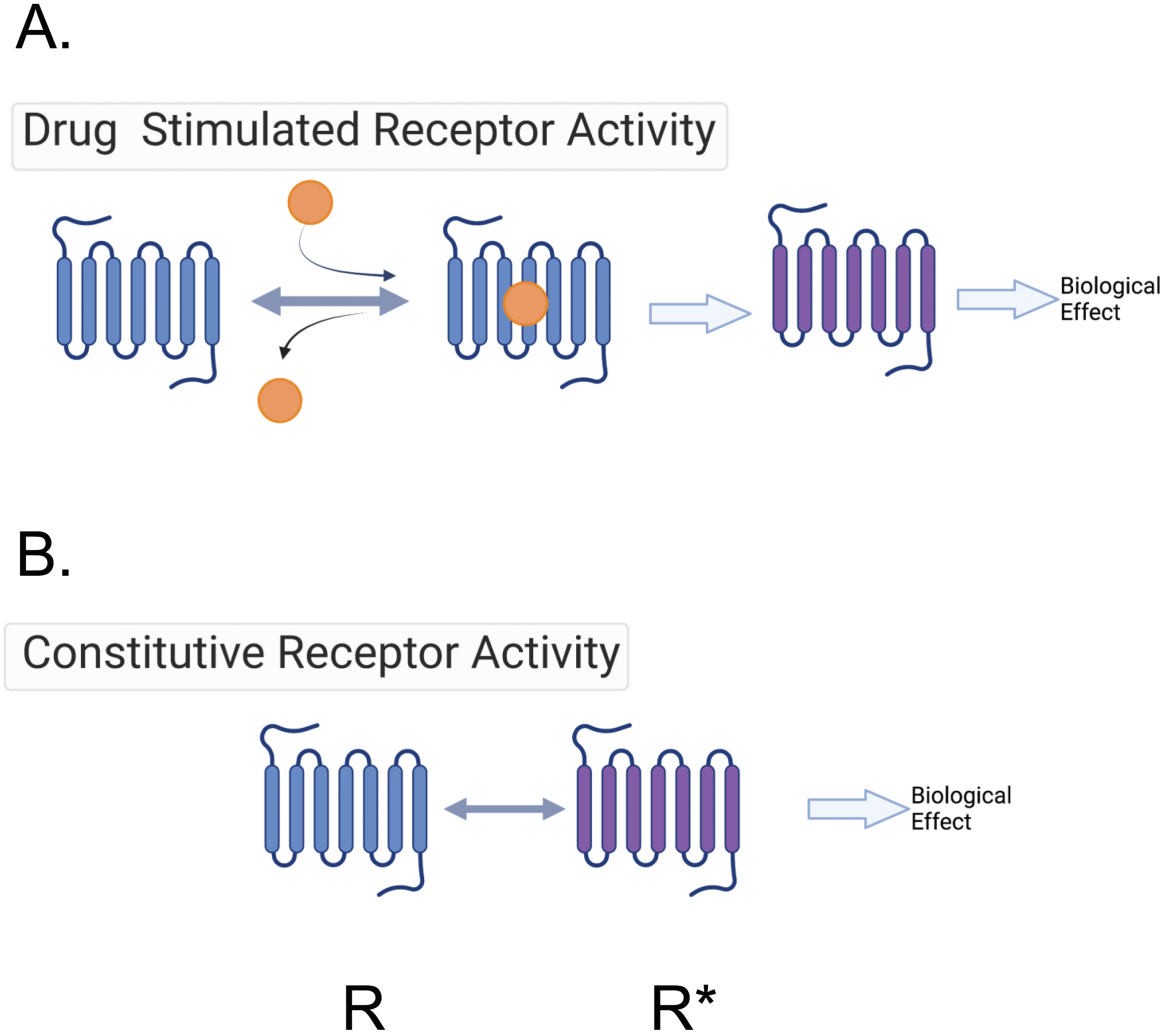
(**a**) Cartoon of basic drug receptor theory. The agonist (orange circle) is in equilibrium with the receptor and can move forward with biological action (change of receptor from blue to purple). (**b**) Depiction of receptor constitutive activity, where receptor can effect biological function in the absence of ligand.

At its essence, drug receptor theory codifies and quantifies how a drug interacts with a receptor to change biological function. Figure 3a illustrates the basic model that a drug binds reversibly to a receptor, obtaining an equilibrium of drug binding to the orthosteric site to form a drug–receptor complex. For high-affinity drugs, the forward reaction of the drug binding to the receptor happens at a faster rate than that of the drug receptor complex falling apart. This working theory does not apply to irreversible or covalently bound drugs (such as phenoxybenzamine) because their actions do not allow for the drug complex to fall apart and a reverse reaction to occur.

Affinity helps determine the potency of a drug. Pharmacologically, potency is measured as the concentration or dose of a drug that is necessary to cause a half-maximal effect (e.g., EC_50_, ED_50_). If a drug has high affinity for a receptor, it stands the chance of being highly potent, or working at very low concentrations, if it also has efficacy, discussed next.

## Agonism: EFFICACY

**Figure 4.**
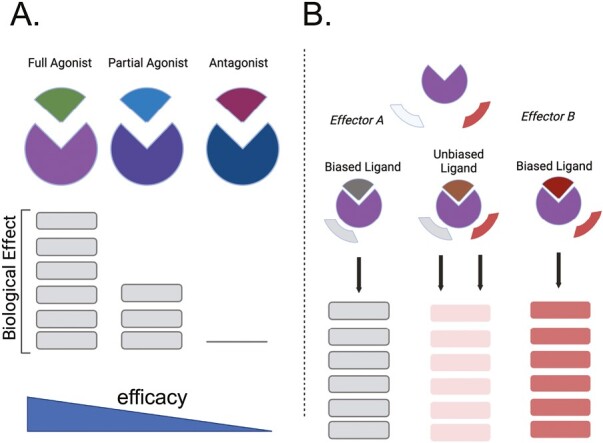
(**a**) Comparison of the magnitude of biological effect (stacked bars) between a full agonist (green), partial agonist (blue) and antagonist (burgundy). Stacked bars compare the magnitude of the biological effect observed in the presence of these agonist. (**b**) Depiction of biased agonism. Different ligands (gray, brown, burgundy), have the ability to elicit a different biological effect based on how they stimulate the signal transduction available to that receptor (effector A, effector B). Stacked bars below are colored differently to indicate that the effect elicited by these three different ligands—ligand biased to effector A, unbiased ligand, biased ligand to effector B—is different in outcome.

**Figure 5.**
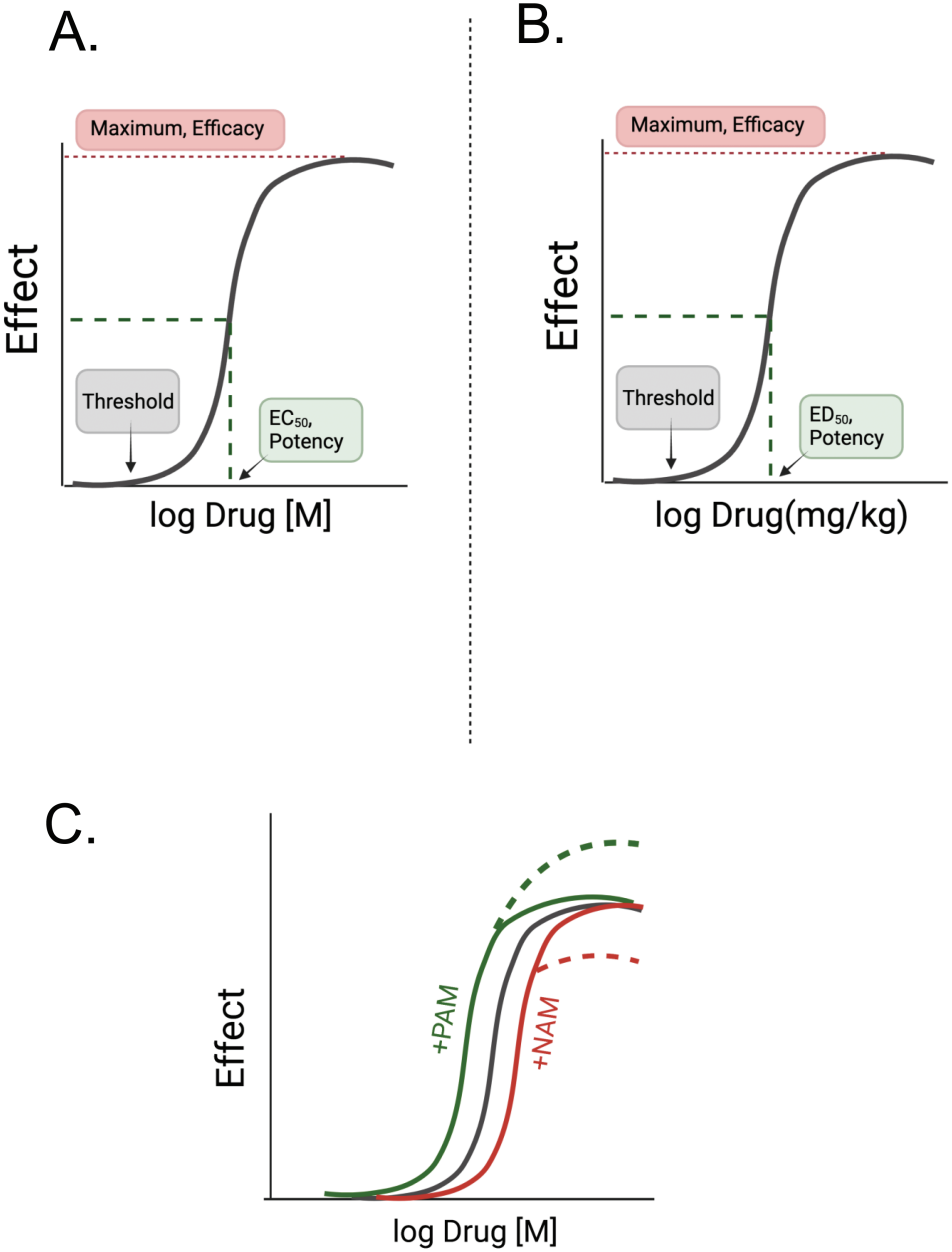
(**a**) Pharmacological parameters (threshold, E**C**50, Maximum) that can be derived from a concentration response curve (typically carried out *in vitro*). (**b**) Pharmacological parameters (threshold, E**D**50, Maximum) that can be derived from a dose response curve (typically carried out *in vivo*). (**c**) Depiction of how a positive allosteric modulator (green; PAM) and negative allosteric modulator (red; NAM) might change a concentration response curve (black line = control curve).

When a drug interacts with the **orthosteric site** of a receptor, this interaction can result in several outcomes. If the drug stimulates a change in receptor function, we say that it has **efficacy.** The ability of a drug to activate a receptor is currently thought to derive from changes in the receptor conformation such that the changed receptor, either directly or indirectly, alters cell function. A direct effect could be the opening of an ion channel in the receptor resulting in changed membrane permeability (e.g., for a ligand-gated ion channel receptor). Alternatively, the activated receptor may cause the response indirectly by interacting with either a transducer (e.g., G proteins) or an effector (e.g., kinase) molecule to mediate the biological signal. A drug that does this would be called an **orthosteric agonist**. In Figure 3a, the activated conformation of the receptor is illustrated by the change in color from blue to purple. If the drug interacts with the orthosteric site but does *not* change the physical structure of the receptor, the function of the receptor does not change. This drug is called an **orthosteric antagonist**. By definition, it has an efficacy (*e* value) of zero. Different drugs may have different abilities to activate a receptor and it is here that the quantitative nature of efficacy is defined. A drug with full ability to activate a receptor, resulting in the maximum effect of the receptor is given a positive efficacy value [which may range from 0 to a large number; efficacy is not necessarily limited to a maximum of 1]. Such a drug that gives a full response in a system is known as a **full agonist**. There are also drugs that cause a response less than that of the full agonist; they are called **partial agonists**. This concept is illustrated in Figure 4a. Figure 5 depicts classic concentration (A) and dose (B) response curves from which potency and efficacy can be assessed. The threshold, as marked in this figure, is the lowest concentration of the drug at which a biological event is observed.

## Allosterism

There are sites on the receptor to which a drug can combine that are different from the orthosteric site; these are called **allosteric sites** (Figure 2b). A drug which interacts with this site and changes the function of the receptor is an **allosteric modulator**.^11,12^ A receptor can have many allosteric sites. The interaction of a drug at these sites fine-tunes the function of a drug binding at the orthosteric site. The allosteric agonist may change the functioning of the orthosteric site in a positive or negative way. Generally, allosteric molecules show the greatest effect in the presence of orthosteric-agonist stimulation, providing fine tuning vs. gross regulation of receptor function. These are defined as PAMs (**positive allosteric modulator**^13^; and NAMs (**negative allosteric modulator**). Allosteric agonists can change either the potency or efficacy of an agonist. The outcome of the promotion or reduction of the actions of an orthosteric agonist is depicted in Figure 5c.

## Constitutive activity and inverse agonism

A multitude of discoveries led to the idea that receptors could possess biological activity in the absence of a cognate agonist, illustrated in Figure 3b. This is called **constitutive activity**. This idea was first supported by data from Cerione *et al*.^14^ using purified β_2_ adrenergic receptors. Some of the strongest evidence for physiologically important constitutive activity exists for the 5-HT_2C_ receptor. 5-HT_2C_ receptor function could be measured *in the absence* of its agonist 5-HT.^15,16^ This means the activity of a receptor is governed by both agonist-*independent* activity—or **constitutive activity**—as well as by that stimulated by an orthosteric agonist. This concept modified drug receptor theory to now suggest that receptors can exist in two states—the R and R* states (Figure 3b)—where R* exemplifies that receptor which has constitutive activity. With recognition of this new function of a receptor, a new class of drugs was named, **inverse agonists**. These drugs are properly called agonists because they stimulate a change in receptor function. They are inverse because their efficacy is negative (*e* = −1 to 0). In this modified theory, drugs which draw the R* to the R are inverse agonists.

In this theory, it is only in systems in which receptors have constitutive activity that inverse agonists cause a measurable effect. If a pathological state is caused by a receptor which has (gained) constitutive activity, for example through a germline mutation, an inverse agonist could be the means to silence the activity of this receptor. A neutral antagonist (with no efficacy) may not be sufficient to inhibit its actions given that a neutral antagonist would not inhibit constitutive activity. Two significant criticisms are made of this concept. First, the possibility always exists that in an experimental system, unknown agonists could activate a receptor and thus this observed activity is not truly agonist independent. Second, most experimental studies done to determine/measure inverse agonism are done in cells that are transfected with the receptor of interest, usually with very high receptor expression. Whether such a system truly reflects the physiological situation is at the heart of this second criticism.

## Biased agonism

Receptor theory changed yet again with the finding that a specific GPCR could be activated by *different* ligands to cause *different* biological outcomes. As illustrated in Figure 4b, we know that receptors can couple to multiple different signal outputs (e.g., G proteins vs. β-arrestins; distinct G proteins—*G*_s_, *G*_q_, or *G*_i_). It is also possible for agonists to cause distinct conformational changes that can bias a receptor towards stimulating one particular pathway vs. another. This idea was formally introduced by Kenakin^17^ and the term “biased agonist” was coined by McKinnon *et al*.^18^ The best established example of this is the G protein vs. β-arrestin bias for GPCRs.^19^ Receptor recruitment of β-arrestin molecules was originally described as a way to stop the signaling of a GPCR, but Luttrell *et al*.^20^ showed in 1999 that β-arrestin, recruited by the activated β_2_ adrenergic receptor, could activate the tyrosine kinase Src.

In theory, a G protein-dependent and/or a β-arrestin-driven signal results from an activated β adrenergic receptor. The signals that emanate from the receptor are not necessarily sequential but may occur in parallel. Also, they may not be activated equally by a given agonist. In terms of efficacy, this means that a drug which is 90% biased towards G proteins would have a higher efficacy for this output measure (effector 1, Figure 4b), and a lower one (10%) at a measure of β-arrestin actions (effector 2). This agonist would be called **G protein-biased**. If only the β-arrestin signaling arm was activated by the agonist, that drug would be β-**arrestin-biased**. In other words, agonists do not have one efficacy value but multiple values, depending on the specific signal transduction pathway downstream of the receptor. Similarly, a receptor signal may be biased toward one G protein or another such as *G*_s_ and cAMP signaling or *G*_q_ and Ca^++^ signaling.^21^ The concept of biased signaling and data to support biased agonism is less well-developed for ion channel receptors. However, for tyrosine kinases, there is increasing data for biased signaling.^22^ Similarly, the concept of selective estrogen receptor modulators (SERMs) is well established as a type of ligand biased for a nuclear receptor.^23^ This nuanced activity of agonists is being used to develop drugs that produce a therapeutic action of a receptor while not causing pathological or adverse actions of the same receptor. This mechanism is particularly relevant to the later discussion of the angiotensin II Type 1 receptor tools.

## Antagonism

Antagonists are drugs which lack the ability to activate a receptor. They do, however, block activation by agonists. Many of the commonly used drugs to treat hypertension are receptor antagonists. The term *neutral antagonist* means that this molecule lacks the ability to reduce constitutive activity nor can it activate a receptor. Such a drug has an efficacy value (*e*) that is zero (0). Pharmacologically, the actions of a neutral antagonist are observed as a rightward shift of the curves.

**Figure 6.**
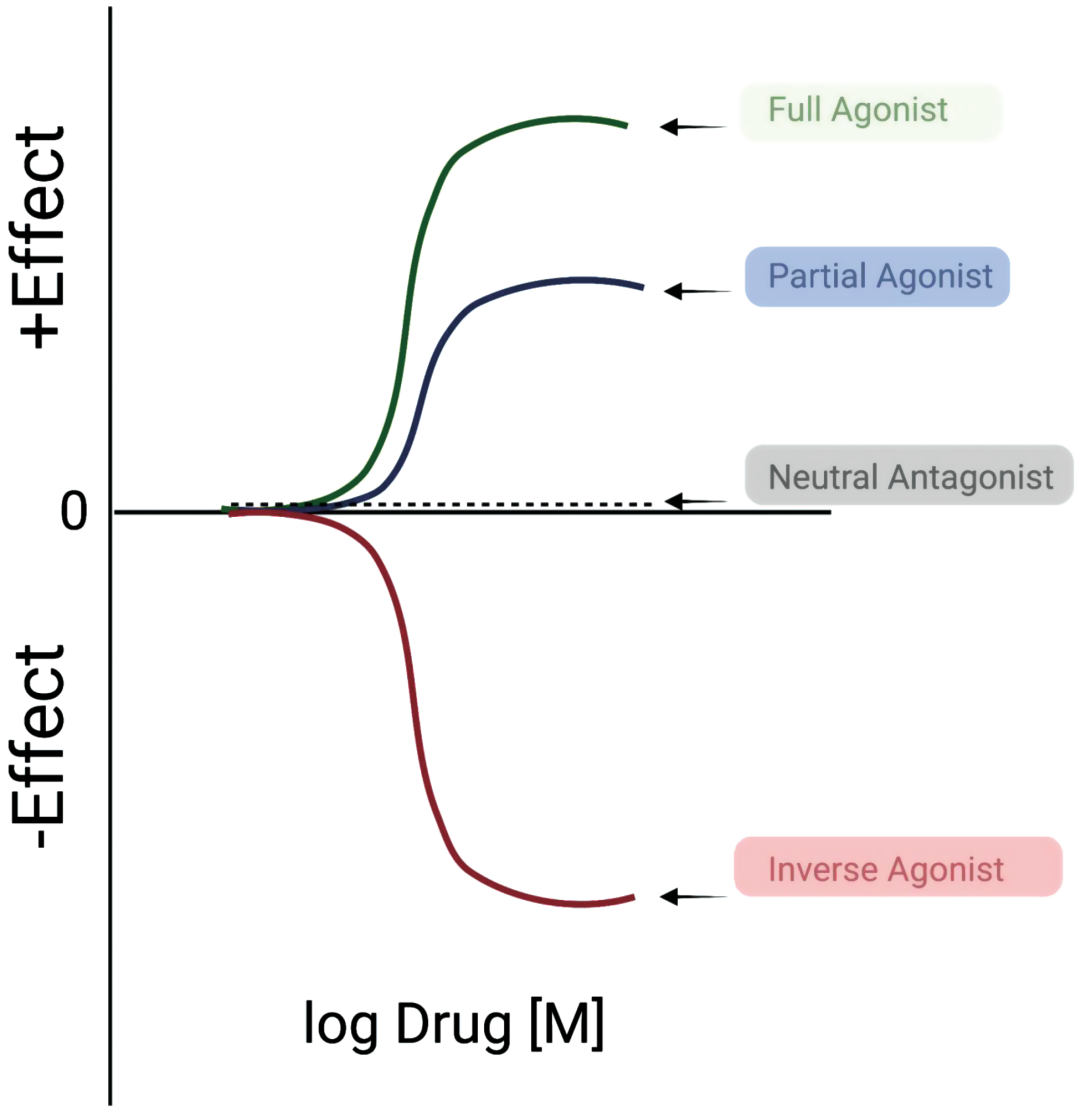
Comparison of the baseline effects of the four classes of ligands described in this review. On the *y*-axis is the effect of these ligands elicited on their own.

Figure 6 compares the major types of drug actions in terms of the biological effect measured with that drug alone.

## Relevance of modified drug theory in hypertension drugs

What does all this mean for hypertension therapeutics? Given that drugs and receptors function in ways not known when many of the hypertension drugs in Figure 1 were discovered, it is important to know whether the drugs used currently possess these new actions. This is especially important relative to potential allosterism vs. orthosterism, and efficacy (inverse, biased agonism). So much more information is available about these concepts for GPCRs vs. other receptors. The lack of discussion of these new concepts for receptors other than GPCRs does not mean non-GPCRs do not possess these new actions, but rather that these characteristics have yet to be observed.

## Harmonized antihypertensive drugs

In 2022, Whelton *et al*.^24^ published the Harmonization of the Hypertension guidelines for multiple societies across the world. In this document, four major drug classes were recommended as first-line therapies. These include AT_1_ receptor antagonists/blockers; angiotensin converting enzyme or ACE inhibitors; thiazide diuretics; and calcium channel antagonists. Of these four major classes, only the AT_1_ receptor is a GPCR. We discuss the primary actions of some of these drugs and the potential “new” pharmacological action of these first-line drugs. This is followed by a discussion of second-line drugs used in the pharmacological treatment of hypertension, many of which target GPCRs. We consulted the publications of leaders in the field in including the drug classes included below.^25–28^ We also consulted the FDA database (June 2023 accession dates; https://www.fda.gov/consumers/free-publications-women/high-blood-pressure) for lists of drugs in each class that are approved for use in the treatment of hypertension. We do not consider drugs that have been combined in a medication.

For first-line therapy, we will not discuss ACE inhibitors or diuretics given that the targets of these drugs and proteins are outside the four recognized receptor subtypes. Nonetheless, Table 1 includes a fairly comprehensive list of first and second-line drugs, with both generic and Trade names.

**Table 1.** Brand names for drugs discussed in this article

Receptor/class	Generic name	Brand names
AT_1_ receptor antagonists	Azilsartan	Edarbi
	Candesartan	Atacand
	Irbesartan	Avapro
	Losartan	Cozaar
	Olmesartan	Benicar
	Telmistartan	Micardis
	Valsartan	Diovan
Calcium channel blockers (dyhydropyridine-type)	Amlodipine	Katerzia, Norvasc
	Felodipine	Plendil
	Nifedipine	Adalat, Procardia
	Isradipine	DynaCirc generic
	Levamlodipine	Conjupri
	Nisoldipine	Sular
Calcium channel blockers (non-dihydropyridine)	Diltiazem	Cardizem, Cartia, Diltzac, Tiazac, Taztia
	Verapamil	Calan, Verelan
Thiazide diuretics	Chlorothiazide	Diuril
	Chlorthalidone	Hygroton
	Hydrochlorothiazide	Microzide, Hydrodiuril
	Metalazone	Zarroxolyn
Angiotensin converting enzyme (ACE) inhibitors	Benzapril	Lotensin
	Captopril	Capoten
	Enalapril	Epaned, Vasotec
	Fosinopril	Monopril
	Lisinopril	Prinivil, Obrelis, Zestril
	Moexipril	Univasc
	Perindopril	Coversyl, Aceum
	Quinapril	Accupril
	Ramipril	Altace
	Trandolapril	Mavick
β_1_ adrenergic receptor antagonists	Acebutolol	Sectral
	Atenolol	Tenormin
	Betaxolol	Kerlone
	Bisoprolol	Zabeta, Concor
	Carvedilol	Coreg
	Labetalol	Trandate
	Metoprolol	Toprol, Lopressor
	Nadolol	Corgard
	Nebivolol	Bystolic
	Pindolol	Visken
	Propranolol	Inderal, InoPran
	Timolol	Blocadren
α_2_adrenergic receptor agonists	Clonidine	Catapres
	Guanfacine	Tenex
α_1_ adrenergic receptor antagonists	Doxazosin	Cardura
	Phenoxybenzamine	Dbenzyline
	Prazosin	Minipress
	Terazosin	Hytrin
Mineralocorticoid receptor antagonists	Eplerenone	Inspra
	Spironolactone	Aldactone
	Finerenone	Kerendia
Dopamine receptor agonists	Fenoldopam	Corlopam
Renin inhibitor	Aliskiren	Tekturna, Rasilez
Glucagon like peptide 1 (GLP1) agonists	Semaglutide	Ozempic, Wegovy, Rybelsus
	Lixisenatide	Adlyxin
Endothelin receptor antagonists	Apricitentan	Not Approved in US

## FIRST LINE HYPERTENSION DRUGS

1 Angiotensin receptor antagonist/blockers (ARBs)

AT_1_ receptor antagonists are one of the newest classes of hypertension drugs (1993) but they were developed prior to the new concepts described above. This is the class for which there is a significant “new” understanding of their mechanism of action. We address biased agonism, inverse agonism, and allosteric modulation.

*FDA-Approved AT*_*1*_ *receptor antagonists:* azilsartan, candesartan, irbesartan, losartan, olmesartan, telmistartan, and valsartan.

*Primary Mechanism of Action:* The primary goal of these drugs is to act as a neutral antagonist to block endogenous Ang II from binding the angiotensin (AT_1_) receptor. This prevents/reduces the hypertensive actions of Ang II.

*New Mechanisms of Actions:*

- Biased Agonism: The differential actions of AngII analogs at the AT_1_ receptor is one of the most fully developed concepts toward the clinical development of biased ligands in the cardiovascular system. AngII can activate both G protein (*G*_q_) and β-arrestin dependent signaling pathways through the AT_1_ receptor to similar extents, thus acting as an unbiased agonist.^29,30^ Surprisingly, a derivative, [Sar1-Ile4-Ile8]-angiotensin II (SII), does not activate G proteins but still induces β-arrestin-dependent signal transduction pathways.^30,31^ Recent studies have defined specific conformational changes in the receptor and ligand that underlie the biased signaling, providing a strong basis for clinical development.^32^ SII was thus one of the first recognized β-arrestin biased agonists. The biotech company Travena built on these findings and developed analogs with improved potency compared to SII. TRV027 and TRV076 are AngII derivatives that lack G protein activation.

Preclinical data in rodent models of hypertension show efficacy. TRV027, given centrally, reduced blood pressure of mouse made hypertensive with the mineralocorticoid deoxycorticosterone acetate.^33^ TRV027 also reduced vessel dilation in an aortic aneurysm study in high-fat-diet-fed ApoE (apolipoprotein E gene)-null mice infused with Ang II.^34^ However, olmesartan produced an even more pronounced effect on vessel cross-sectional area and elastin content. TRV027, as an antagonist of the *G protein*-coupled AT pathway which while promoting *β-arrestin* pathways, improved cardiac structure and function in a genetic mouse model of dilated cardiomyopathy.^35^

In the Phase IIb clinical trial in acute heart failure (BLAST-AHF), TRV027 unfortunately did not meet clinical endpoints.^36^ A *post hoc* subgroup analysis^37^ gave indications there may be some clinical benefit in patients with higher blood pressures but this will need to be examined further. Based on the preclinical findings, other indications such as resistant hypertension, may be worth exploring. Overall, activating the β-arrestin signaling pathway through the AT_1_ receptor while antagonizing the G protein arm of signaling may prove useful in the appropriate therapeutic context.

- *Inverse agonism*: Several of the FDA-approved AT_1_ receptor antagonists possess inverse agonist activity. Specifically, the biphenyltetrazole antagonists have robust inverse agonism. This is exemplified by one of the first AT_1_ receptor antagonists discovered, losartan, as well as candesartan, olmesartan, irbesartan, and valsartan.^38^ Structural and modeling studies support that these clinically used drugs have inverse agonist character. This inverse agonism could contribute to their *in vivo* therapeutic effect. In hindsight, these drugs are brilliant in that they not only block the AT_1_ receptor but may also inhibit any constitutive activity of the AT_1_ receptor. The inverse agonism could also inhibit the beneficial β-arrestin coupled pathways activated through the AT_1_ receptor, but that would move the AT_1_ receptor into a state where G protein signaling also cannot carry out the detrimental effects of the AT_1_ receptor.
- *Allosterism:* The study of allosterism at the AT_1_ receptor has recently begun in relation to agonistic auto-antibodies. Singh et al raised the idea that these auto-antibodies bind to a site different than the orthosteric binding site of Ang II.^39^ They discovered a druggable allosteric pocket in the AT_1_ receptor as well as compounds that could negatively modify the biological function of the AT_1_ receptor. These compounds reduced AngII-induced contraction of the isolated artery by acting as NAMs of the AT_1_ receptor. By contrast, a peptide known as a hemorphin (specifically LVV-hemorphin-7) serves as a PAM at the AT_1_ receptor.^40^

2. Calcium channel antagonists/blockers (CCBs)

The primary target of these antagonists is the vascular L-type Calcium channel, also known as Cav1. This channel, when open, permits the movement of extracellular calcium from outside to inside the cell, activating vascular contraction (and heart inotropism and chronism).

*FDA-Approved Drugs:* amlodipine, ailtiazem, felodipine, isradipine, levamlodipine, nifedipine, nisoldipine, and verapamil.

*Primary Mechanisms of Action:* The L-type channel inhibitor nifedipine (a dihydropyridine), as well as the phenylalkylamine verapamil, bind to the alpha subunit of the channel and stabilize the inactive form of the channel.^41^ It would not be wrong to call these drugs inverse agonists in that they bring this receptor from a state of action (open; R*) to one of inactivity (closed; R).

*New mechanisms of action:* The L-type calcium channels, like many voltage-gated ion channels, have a complex conformational repertoire including open (active) and closed and inactivated (both inactive). The inactivated state represents a refractory (or desensitized) state where membrane depolarization will not activate the channel. There are subtle functional differences between the dihydropyidines (e.g., amlodipine) and the phenylalkalamines (e.g., verapamil) including differences in the voltage dependence of blockade.^42^ Structural analysis indicates that dihydropyridines act allosterically to modulate the ion selectivity filter for Ca^++^ permeability.^43^ The binding site for dihydropyridines is located in the membrane on the lipid-facing surface of the channel pore region, between two subunits. This results in an asymmetric conformation of the selectivity filter, causing incompletely hydrated Ca^++^ to block the pore. On the other hand, the phenylalkylamine verapamil binds directly to the central cavity of the ion permeation pore at the selectivity filter, physically blocking ion-conduction. There does not appear to be any specific signaling bias (e.g., alteration of the selectivity of ion permeability) but the mechanistic differences related to voltage sensitivity do appear to contribute to the selectivity of dihydropyridine drugs for vascular smooth muscle, which is more depolarized than cardiac muscle.

## SECOND LINE HYPERTENSION DRUGS

The following classes are considered second-line therapy for the treatment of hypertension. More of these classes of drugs are GPCRs and thus better known relative to their potential new actions.

1. Beta adrenergic receptor antagonists/blockers

The β adrenergic receptors are GPCRs, and it is specifically the β_1_ adrenergic receptor in the heart that is the target of this class of antihypertensives.

*FDA-Approved Drugs:* acebutolol, atenolol, betzxolol, bisoprolol, carvedilol, labetalol, metoprolol, nadolol, nebivolol, pindolol, propranolol, and timolol.

*Primary Mechanism of Action*: By antagonizing the ability of endogenous norepinephrine to stimulate predominantly the β_1_ adrenergic receptor that increases heart rate and force, these antagonists serve to lower the work of the heart. They can also modify renin release from the kidney. They antagonize the orthosteric bindings site of norepinephrine at this receptor.

*New Mechanisms of Action:*

- Inverse Agonism: A number of related but non-FDA-approved antagonists have been tested in *in vitro* experiments to determine inverse agonism in cells transfected with multiple species of the β_1_ adrenergic receptor. This includes bucindolol and carazolol. The FDA-approved carvedilol and propranolol demonstrated inverse agonism, while bisoprolol and metoprolol were inconsistent in outcome based on the assay used.^44^ It is, however, unknown whether these specific pharmacological actions contribute to the effect in humans.
- Biased Agonism: Carvedilol has been reported to act as a β-arrestin biased agonist,^45^ as has nebivolol.^46^ These drugs would inhibit G protein activation, to which elevated heart rate is typically attributed. They would also activate β-arrestin signaling or at least not inhibit it. The importance of these effects clinically is not clear, especially in light of the pharmacological actions of carvedilol.

2. Alpha2 adrenergic receptor agonists

This is a discrete class of drugs that have been used since ~1965.

*FDA-Approved Drugs:* clonidine, guanfacine.

*Primary Mechanism of Action:* This class of drugs has been used almost exclusively to inhibit the central drive of blood pressure by stimulating inhibitory presynaptic α_2_ adrenergic receptors, ultimately leading to a reduction in sympathetic nervous system drive. This is thought to be mediated predominantly through inhibitor G protein mechanisms (Gi/o signaling).

*New Mechanisms of Action:* Clonidine can also activate G_s_ and β-arrestin biased pathway through human α_2a_^47^ or α_2C_^48^ adrenergic receptors, recombinantly expressed in Chinese Hamster Ovary cells. While guanfacine was not tested, guanabenz also showed the ability to activate both G_s_ and β-arrestin pathways. It is unknown whether these other signal transduction pathways contribute to the antihypertensive effects of these two drugs.

3. Alpha1 adrenergic receptor antagonists

This class of drugs are recognized as effective antihypertensive medications.

*FDA-Approved Drugs:* doxazosin, phenoxybenzamine, prazosin, terazosin.

*Primary Mechanism of Action:* These antagonists reduce total peripheral resistance by antagonizing the α_1_ adrenergic receptor that receives stimulation from the sympathetic nervous system, the endogenous agonists of which is norepinephrine and epinephrine.

*New Mechanisms of Action:* Many of the above antagonists have been investigated for inverse agonism. *In vitro* studies support that at the least prazosin and terazosin have moderate to strong inverse agonism.^44^ By contrast, phenoxybenzamine exhibits irreversible blockade by binding covalently to the receptor.

4. Mineralocorticoid receptor antagonists

While spironolactone has been used since ~1960, eplerenone, approved in 2003 by the FDA, has become a mainstay in treatment.^49–51^ Finrenone was approved in 2021. These drugs have been particularly important in determining whether resistant hypertension is mineralocorticoid-based.

*FDA-Approved Drugs:* eplerenone, finerenone, spironolactone.

*Primary Mechanism of Action:* These drugs block the ability of the mineralocorticoid hormone aldosterone to stimulate sodium and potassium uptake in epithelial cells in the kidney to effect a natriuresis that can lower blood pressure. Non-epithelial site of actions of aldosterone, and thus these antagonists, are also recognized.

*New Mechanisms of Action:* The classical mineralocorticoid receptor is a nuclear receptor. Ligand bias has been well recognized for the nuclear estrogen and androgen steroid hormone receptors in the guise of selective estrogen receptor modulators (SERMs)^52^ and selective androgen receptor modulators (SARMs).^53^ This may be mediated in part by selective recruitment of co-activator or co-repressor proteins to the transcriptional complex. In addition, steroid ligands and their synthetic agonist analogs and antagonists can also mediate non-nuclear or non-genomic effects through membrane-localized actions either of the classical nuclear hormone receptor (estrogen receptor; ER or mineralocorticoid receptor; MR) or possibly through distinct receptors (ER-beta or the G protein-coupled estrogen receptor GPER).^54^ For aldosterone, there is evidence that both the classical MR and non-MR receptors can carry out effects of aldosterone that are faster than nuclear-based effects.^50,55^ Indeed, there is somewhat controversial literature about the role of GPER, either direct or indirect, in the actions of aldosterone in the cardiovascular system.^56^

For the more recently approved MR antagonists, finerenone, mechanistic, and preclinical studies show a unique inverse agonist activity for finerenone vs. eplerenone.^57^ In addition, there may be biased recruitment of transcriptional co-activators and co-repressors. With respect to clinical significance, there were differences between eplerenone and finerenone on the development of cardiac fibrosis after isoproterenol infusion. Both MR antagonists significantly inhibited the isoproterenol-mediated increase in left ventricular mass. However, isoproterenol-induced cardiac fibrosis and inflammation were blocked by finerenone, whereas, eplerenone had no significant effect. Whether either the inverse agonism or co-factor recruitment bias is the mechanism of this unique benefit of finerenone should be assessed in further studies.

5. Dopamine receptor agonists

This is a niche class of antihypertensive drugs.

*FDA-Approved Drugs*: fenoldopam.

*Primary Mechanism of Action:* Fenoldopam’s use has been largely restricted to hypertensive crises.^58^ Its mechanism is somewhat unique in that it activates dopamine D_1_ receptors that reduce vascular resistance primarily in the kidneys. The D_1_ receptor is a GPCR.

*New Mechanisms of Action:* Evidence suggests that the D_1_ receptor can act in a biased fashion, and that fenoldopam may be somewhat β-biased.^59^

## WHY NEW ANTIHYPERTENSIVES?

The sheer fact that resistant and, more so, refractory hypertension exists is evidence that new drugs for the treatment of hypertension need to be discovered and brought to the clinic.^27,28^ All the pathways described above cannot be the totality of means by which blood pressure is regulated. Moreover, the discouraging knowledge that blood pressure control is regressing in the United States makes it all the more important to have different therapeutic agents to which to turn clinically.^60–63^ The sobering problem of these three hypertension R’s—regression, resistance, and refractoriness—are impetus for filling in the blanks in Figure 1 with new discoveries. This certainly includes new classes of drugs which interfere with the mechanisms described above. It also includes taking *better* or smarter advantage of the different ways to manipulate receptor function to make new therapeutics. The drug classes described below are not (yet) FDA-approved for use in treating hypertension.

*Glucagon-like peptide 1 (GLP1) agonists*: A beautiful review of this peptide is in reference.^64^ GLP-1 potentiates glucose-dependent insulin secretion while suppressing glucagon secretion in response to nutrients. As such, agonists of the GLP-1 receptor (GLP-1R), exemplified by semaglutide and lixisenatide, are being developed for the treatment of type 2 diabetes and obesity. GLP-1 receptors are GPCRs and thus they have the potential to demonstrate inverse agonism, biased agonism, and allosteric modulation. Indeed, new peptide and small molecule agonists of the GLP-1R include full agonists, biased agonists, and allosteric modulators.^65^ Song *et al*.^65^ reported that the human GLP-1R possesses negative allosteric modulation sites. The science to separate out the effect of the weight loss caused by these drugs and the reduction of blood pressure is not yet clear. Such an untangling of cause and effect becomes even more complicated in patients comorbid for diseases that present together frequently: diabetes, hypertension, kidney disease, obesity. A few reviews of the literature speak to the modest ability of these inhibitors to lower blood pressure.^66–68^

*Endothelin Antagonists*: Endothelin-1 (ET-1) is a potent pro-hypertensive agent and effects most of its actions through the GPCRs ET_A_ and ET_B_. Aprocitentan, a dual ET_A_ and ET_B_ receptor antagonist, appears to have struck the optimal balance of blockade of these two receptors (16:1 for A vs. B). The PRECISION clinical trials support the use of this antagonist for resistant hypertension.^69^ Xu *et al*.^70^ review ET-1 and ET receptors in the context of aprocitentan. Interestingly, evidence exists that ET receptors possess biased signaling^71^ but it is not known if drugs like aprocitentan take advantage of these new receptor modalities.

## PERSPECTIVES AND CONCLUSIONS

- Receptor theory evolves and continues to do so along-side antihypertensive drug development. There is a seeming advantage to use these new theories in the deliberate development of novel antihypertensives.
- The existence of the three R’s—regression, resistance, and refractoriness demands new developments. While this review does not cover potential new classes of therapeutics (antibodies, RNA therapies, non-pharmacologic, etc.), it provides evidence that there is room within the small molecule world to develop new therapeutics that act with new mechanisms.
